# UVB Radiation as a Potential Selective Factor Favoring Microcystin Producing Bloom Forming Cyanobacteria

**DOI:** 10.1371/journal.pone.0073919

**Published:** 2013-09-13

**Authors:** Yi Ding, Lirong Song, Bojan Sedmak

**Affiliations:** 1 Key Laboratory of Algal Biology, Institute of Hydrobiology, Chinese Academy of Sciences, Wuhan, China; 2 University of the Chinese Academy of Sciences, Beijing, China; 3 Department of Genetic Toxicology and Cancer Biology, National Institute of Biology, Ljubljana, Slovenia; University of New South Wales, Australia

## Abstract

Due to the stratospheric ozone depletion, several organisms will become exposed to increased biologically active UVB (280–320 nm) radiation, not only at polar but also at temperate and tropical latitudes. Bloom forming cyanobacteria are exposed to UVB radiation on a mass scale, particularly during the surface bloom and scum formation that can persist for long periods of time. All buoyant species of cyanobacteria are at least periodically exposed to higher irradiation during their vertical migration to the surface that usually occurs several times a day. The aim of this study is to assess the influence on cyanobacteria of UVB radiation at realistic environmental intensities. The effects of two UVB intensities of 0.5 and 0.99 W/m^2^ in up to 0.5 cm water depth were studied *in vitro* on *Microcystis aeruginosa* strains, two microcystin producing and one non-producing. After UVB exposure their ability to proliferate was estimated by cell counting, while cell fitness and integrity were evaluated using light microscopy, autofluorescence and immunofluorescence. Gene damage was assessed by TUNEL assay and SYBR Green staining of the nucleoide area. We conclude that UVB exposure causes damage to the genetic material, cytoskeletal elements, higher sedimentation rates and consequent cell death. In contrast to microcystin producers (PCC7806 and FACHB905), the microcystin non-producing strain PCC7005 is more susceptible to the deleterious effects of radiation, with weak recovery ability. The ecological relevance of the results is discussed using data from eleven years’ continuous UVB radiation measurements within the area of Ljubljana city (Slovenia, Central Europe). Our results suggest that increased solar radiation in temperate latitudes can have its strongest effect during cyanobacterial bloom formation in spring and early summer. UVB radiation in this period may significantly influence strain composition of cyanobacterial blooms in favor of microcystin producers.

## Introduction

Cyanobacteria evolved in an extreme environment under anoxic conditions, high temperature, large variations of available nutrients and strong solar radiation including UV. These are major adverse factors that influenced their early life on Earth [1]. For these reasons the current competitive strategies of cyanobacteria should also be considered in the framework of their morphophysiological adaptations from an evolutionary aspect. At the end of the last century depletion of the ozone layer that absorbs the harmful radiation from the sun increased the risk of exposure of aquatic organisms to biologically effective UVB radiation. Current best estimates suggest that slow recovery of the ozone layer may be expected only during the next half of the 21^st^ century [2] and that it could take decades before the ozone layer recovers to pre−1980 values [3]. Freshwater ecosystems, especially those in mid to low latitudes, may be affected to a much higher degree by the persisting fluxes of UV radiation [4]. Thus, in relatively shallow freshwater bodies, climate change and stratospheric ozone depletion may act synergistically to increase the exposure of organisms to UVB radiation.

The impacts of harmful UVB radiation in the water ecosystem are restricted to the upper part of the photic layer. The most vulnerable are buoyant organisms, with special emphasis on photoautotrophs that exploit light as their main source of energy. Bloom forming cyanobacteria are among the most numerous phytoplanktonic species frequently exposed on the water surface. Cyanobacterial species that possess an active buoyancy regulation mechanism appear at the water surface several times a day during their vertical migration through the water column and can even persist at the very surface for several hours a day, forming surface blooms and scums. Bloom forming cyanobacteria lacking this type of regulation are in this respect totally dependent for their existence on water mixing activity. Eutrophication, as a consequence of human activities, increasingly accelerates the incidence of harmful cyanobacterial blooms under stable climate conditions [5]. Given that the rising temperatures favor cyanobacterial dominance, Paerl and Huisman [6] claim that there is a link between global warming and the worldwide increasing incidence of harmful cyanobacterial blooms in both their abundance and frequency. Cyanobacterial blooms are a global problem for a great number of reasons, with microcystin (MC) production being the main cause of human fatalities [7]. Ozone depletion, with consequent increased UVB radiation at ground level, is an additional environmental factor that could alter the phytoplankton composition in surface waterbody ecosystems.

Several studies have demonstrated that UVB radiation induces severe damages to cyanobacteria. Genetic material and the photosystem are the main targets of radiation products. The two most mutagenic and cytotoxic DNA lesions, cyclobutane pyrimidine dimers and pyrimidine pyrimidone photoproducts [8] together with reactive oxygen species (ROS) [9] are such examples. Recent experimental data indicate that microcystin plays an important role in producing cyanobacteria under stress conditions [10]. In their attempt to clarify the biological role of microcystin, Zilliges and coworkers (2011) propose this cyanopeptide as protein-modulating metabolite and protectant against oxidative stress. They demonstrated that microcystin binding to several enzymes of the Calvin cycle, phycobiliproteins and two NADPH-dependent reductases is strongly enhanced under high light and stress conditions [11].

Our efforts are directed to understanding how UVB radiation influences cyanobacteria and cyanobacterial bloom formation and composition in an environment of increasing water eutrophication. The aim is to establish how, and to what extent if any, harmful UVB radiation can influence the global dominance of cyanobacteria.

## Materials and Methods

### Strains and Cultivation

Three unicellular *Microcystis aeruginosa* strains were used. Two axenic *M. aeruginosa* strains - the microcystin non-producing (MC non-producing) PCC7005, the microcystin producing (MC producing) PCC7806 from Institute Pasteur (Paris, France) - and the FACHB905 (MC producing) isolated from Dian Lake (Dianchi, Yunnan Province China) (Chinese Academy of Science Collection). The experiment was divided into two groups with appropriate controls. All strains were grown at 25°C and maintained under sterile conditions in 100 ml flasks in 50 ml BG-11 medium exposed to daylight. The first group (PAR-acclimated) was removed from the photosynthetically active radiation (PAR) at 10 a.m., exposed to UVB for six hours at lower intensity 0.5 W/m^2^, and then returned to the 24 h solar day/night cycle. The second group (Dark-acclimated) was dark acclimated for 24 h, exposed to UVB radiation and maintained in complete darkness for the following 48 h.

The experiment was repeated using only two PAR-acclimated cyanobacterial strains (MC non-producing PCC7005 and MC producing PCC7806) exposed to a higher (0.99 W/m^2^) UVB intensity for three hours and returned to the 24 solar day/night cycle.

The cell concentration used in the experiments was 5×10^6^ cells ml^−1^.

### UVB Exposure and Cell Sampling

**Figure 1:**
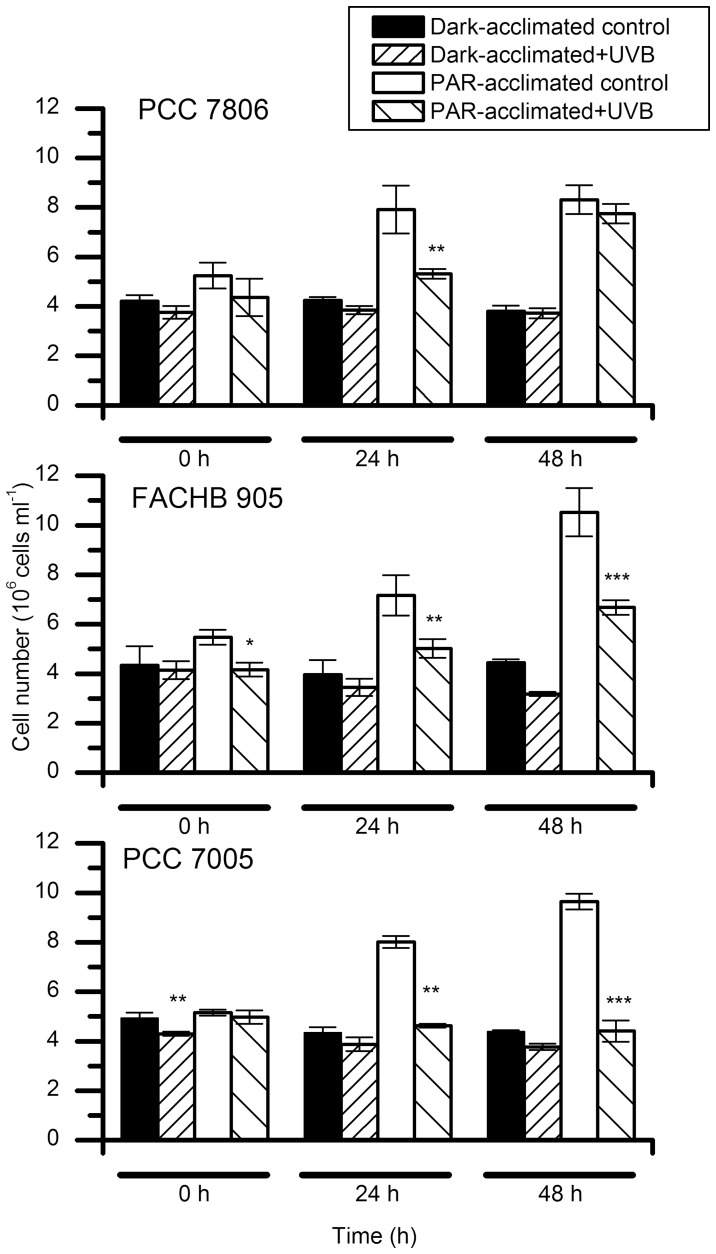
Data from continuous measurement of environmental UVB radiation in Ljubljana city (Central Europe). Panel A. Daily fluctuations in average UVB intensity for three selected months in the year with the lowest (1998) and in the year of the highest cumulative UVB radiation dose (2003). The dashed horizontal line indicates the average UVB intensity used for 6 hours exposure in *in vitro* experiments (Automatic Amp Station Ljubljana-Bežigrad). The city of Ljubljana is situated in Central Europe (latitude 46°3′5″ N, longitude 14°30′20″ E) at 291 m (954 ft) above sea level. Panel B. Average solar UVB hourly doses in spring (April), summer (July) and autumn (October) for the years 1998 and 2003. The dashed horizontal line indicates the dose (1800 J/m^2^) used in *in vitro* experiments. Zero values derived from days without solar irradiation are omitted.

Both UVB intensities used in our experiments were chosen on the basis of environmental measurements. In late spring during their growth season cyanobacteria are frequently exposed to continuous UVB radiation reaching and exceeding intensities of 0.5 W/m^2^, while in summer the relative surface blooms can be exposed for several hours to intensities higher than 1 W/m^2^ (Figure 1).

Cell counts were determined with a hemocytometer (Blaubrand, Germany). Each of the three strains in covered plastic Petri dishes (90 mm diameter)was separately exposed in triplicate to UVB radiation in up to 0.5 cm BG-11 medium depth, at a concentration of 5 ×10^6^ cells ml^−1^ in 20 ml BG11 medium. The plastic cover is an effective filter for UVC radiation [12]. A Q-Panel UV-B 313 fluorescent lamp (Cleveland, OH, USA) was used as the UVB source. The radiation was measured using a Radiometer (RM22, Dr. Gröbel Elektronik GmbH, Germany). Control groups were not exposed to UV radiation and were either maintained on the natural solar day/night cycle (PAR-acclimated) or kept in darkness (Dark-acclimated). Samples were collected immediately after UVB exposure (time 0), 24 and 48 h after irradiation. The cyanobacterial cells irradiated with the higher UVB intensity (0.99 W/m^2^) were under observation for the next twenty days exposed to PAR.

### Terminal Deoxynucleotidyl Transferase Labeling (TUNEL) Assay

TUNEL assays were carried out with an In Situ Cell Death Detection Kit, Fluorescein (Roche Diagnostics, Cat.No.11 684 795 910, Mannheim, Germany). Control cells and cell samples at 0, 24 and 48 h after UVB irradiation (0.51 W/m^2^ for six hours) were collected. They were fixed at room temperature for 1.5 h with 2% paraformaldehyde in PBS, washed with PBS and permeabilized for 20 min at 4°C in solutions containing 0.1% Triton X-100 and 0.1% sodium citrate. The labeling and signal conversions were carried out according to the manufacturer’s instructions. Negative controls were treated as described above but labeled only with the label solution (without terminal transferase). Positive controls consisted of permeabilized cells pretreated with DNase I (Invitrogen, Cat.No.18047-019) for 20 min prior to labeling with the TUNEL reaction mixture. Labeled samples were analyzed under a fluorescence microscope (Olympus BX51, Japan) at an excitation wavelength in the range of 450–500 nm, and results were processed using the Image-Pro Express 6.0 software program. Representative images were taken after analysis of at least 500 cells per sample.

### SYBR Green Staining

Control cells and cells collected after UVB irradiation were washed with BG-11, fixed in 3.7% paraformaldehyde in BG-11 for 60 min at room temperature, washed again three times with BG-11 medium and permeabilized in BG-11 medium containing 0.1% Triton X-100 (v/v) (Merck, Germany) for 20 min. The cells were then incubated with SYBR green (Sigma, USA) (1∶500 in BG-11) for 15 min at room temperature in the dark. Images were recorded on an epifluorescence microscope Nikon Eclipse T300.

### Immunostaining of the Cytoskeletal Framework

Cells were prepared and immunostained as reported [13]. The target in *M. aeruginosa* strains was the Unnamed protein product (accession number CAO86402) [14].

### Phase Contrast and Epifluorescence Microscopy

Pigment autofluorescence was recorded using a G-2A filter with excitation from 541 to 551 nm (bandpass 546 CWL, dichromatic mirror cut-on 565 nm, barrier filter cut-on 590 nm). The cytoskeleton framework and the SYBR Green fluorescence were recorded using a B-2A filter with excitation in the blue light region from 450 to 490 nm (bandpass, 470 CWL, dichromatic mirror cut-on 500 nm, barrier filter cut-on 515 nm). The images were recorded on a Nikon Eclipse T300 microscope with Super high-pressure mercury lamp power supply HB-10103AF using an oil immersion objective, magnification 1000X. The images were processed using NIS-Elements D (Nikon, Japan) software. Representative images of control cells and cells after UVB irradiation were taken after analysis of at least 500 cells per sample.

### Sedimentation

The cyanobacterial strains were divided into two groups with related controls and irradiated for 6 hours at an intensity of 0.51 W/m^2^ as described above. After UVB exposure the PAR-acclimated group was allowed to settle for 48 h in 100 ml flasks exposed to natural 24 h solar day/night cycle. The Dark-acclimated group was kept in the dark for 24 h before UVB exposure and returned to complete darkness for the next 48 h.

### Assessment of Sedimentation and Proliferation

Each strain from each group was distributed equally into three culture flasks that were then kept undisturbed for 24 or 48 hours. The cell concentrations of the PAR- and Dark-acclimated groups were assessed by cell counting, first by taking samples from the middle of the water column of the undisturbed culture and then after thorough mixing of the culture. In this way the total cell concentrations for individual sedimentation periods and the average cell concentrations in the suspensions were both obtained. Sedimentation was quantified by subtracting the average cell concentration in suspension from the total cell concentration after mixing.

### Measurement of Environmental UVB Radiation

The data for UVB radiation were obtained from the Slovenian Environmental Agency. Raw data for two years (1998 and 2003) from continuous 24 hours UVB radiation measurements (the automatic Amp Station Ljubljana-Bežigrad) were processed (Figure 1). The city of Ljubljana is situated in Central Europe (latitude 46°3′5″ N, longitude 14°30′20″ E) at 291 m (954 ft) above sea level and has an Oceanic climate (Köppen climate classification “Cfb”) with continental characteristics. 1998 was chosen as the year with the lowest (cumulative UVB dose 2.5 10^6^ J/m^2^) and 2003 as the year with the highest UVB radiation (cumulative UVB dose 3.0 10^6^ J/m^2^) at ground level. The average daily UVB intensities for the months of April (spring), July (summer) and October (autumn) were calculated (Figure 1, Panel A), together with the UVB doses for hypothetical average individual days in the same months in both years. The highest UVB intensities were also determined. Data for the days without sun were omitted in the figures showing the hourly distribution of the UVB dose of the hypothetical day for single months (Figure 1, Panel B).

### Data Analysis

All experiments were performed in triplicate. Data are presented as means ± standard deviations and analyzed using Microcal Origin Software (Version 8.0, Microcal Software Inc. Northampton, MA, USA). Significant differences between control and treated samples were determined by one-way ANOVA followed by a Tukey’s test. Differences were considered to be significant when P<0.05. UVB radiation data were presented using GraphPad Prism 5 (GraphPad Inc., USA).

## Results

### Influence of UVB Radiation on Cell Proliferation

**Figure 2:**
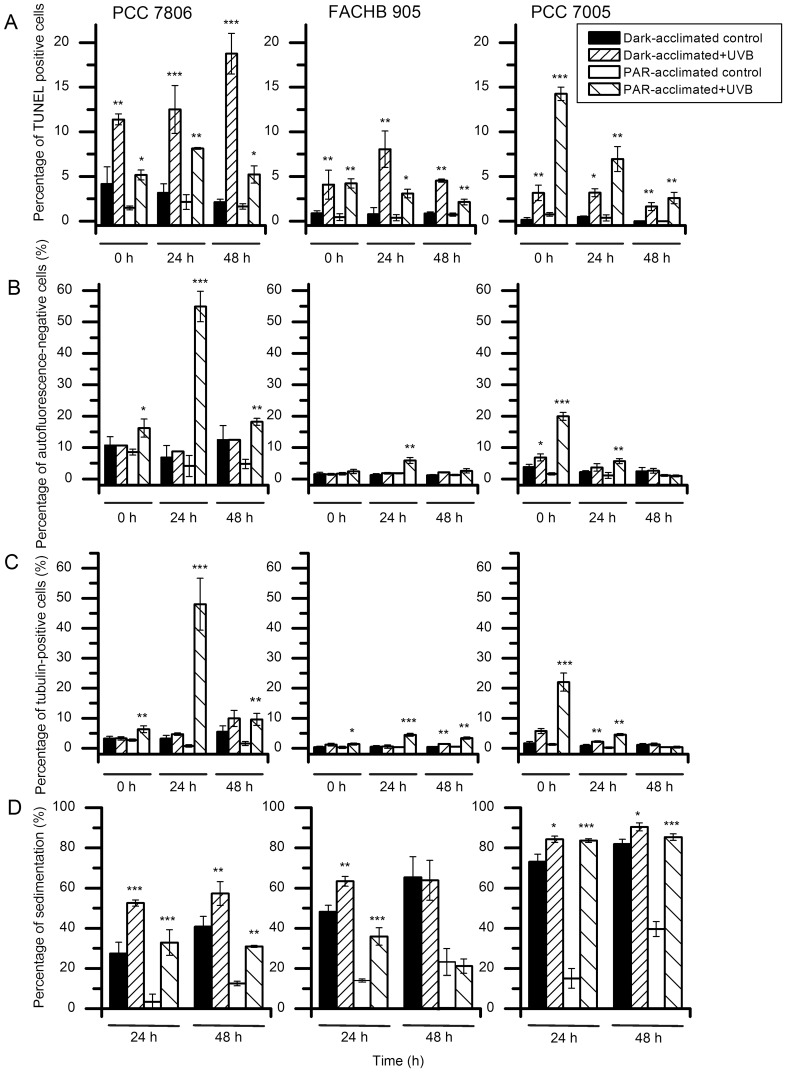
Proliferation of the three *M. aeruginosa* strains after a six-hour exposure to UVB radiation at intensity of 0.51 W/m^2^. (*p<0.05, **p<0.01, ***p<0.001).

After PAR exposure all three strains in controls were able to proliferate normally and approximately doubled their concentration in 48 h, while the Dark-acclimated group was unable to proliferate in the absence of light (Figure 2). After UVB exposure (0.51 W/m^2^ for six hours) lower cell count was observed in all three PAR acclimated strains where both MC producers (PCC7806 and FACHB905) recovered from the stress induced and resumed proliferation. On the contrary the MC non-producing PCC7005 strain was unable to increase in cell number in the period of 48 hours after UVB exposure (Figure 2). The cell count for treated PCC7005 strain remained at the starting level throughout the experiment. There were no significant changes in cell number in the Dark acclimated MC producing strains after UVB exposure although FACHB905 strain showed a downward trend in cell concentration. In contrast Dark-acclimated MC non-producing PCC7005 strain exhibited a significant decrease in cell number immediately following UVB exposure (Figure 2).

**Figure 3:**
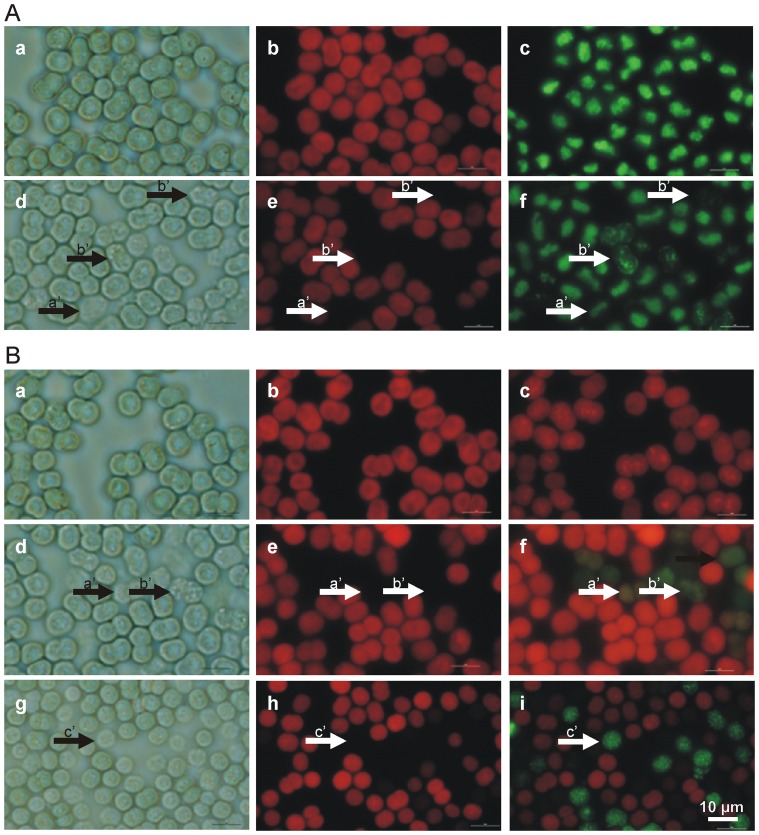
Study of both MC non-producing (PCC7005) and MC-producing (PCC7806) *M. aeruginosa* strains exposed to UVB radiation for three hours at the intensity of 0.995 W/m^2^. Panel A. Upper row, control MC non-producing PAR acclimated PCC7005 strain, middle row - PCC7806 strain exposed to UVB radiation and lower row – PCC7005 strain exposed to UVB radiation. Microphotographs of the same area of *M. aeruginosa* cells taken under light microscopy (Images a, d and g), autofluorescence (Images b, e and h) and SYBR Green DNA staining (Images c, f and i). Control cells in the upper row show normal cell morphology (Image a) with homogeneous autofluorescence (Image b), and compact nucleoids (Image c). Middle row - Twentyfour hours after UVB exposure the majority of PCC7806 cells udergo the process of lysis (Image d), with reduced autofluorescence (Image e). The degree of autofluorescence was estimated by using exposure times (Image b 83 ms; Image e 83 ms; Inset Image e 833 ms and Image h 917 ms). Inset in Image e is a microphotograph of the same area of cells taken at an extended exposure time. SYBR Green staining reveals complete degradation of the genetic material 24 hours after exposure expressed also as a weak signal (Image c – control cells, exposure time 250 ms; Image f – PCC7806, 250 ms and Image I – PCC7005, 917 ms). Scale bar = 5 µm. Panel B. Macroscopic representation of the three *M. aeruginosa* cell strains exposed to UVB radiation for three hours at an intensity of 0.995 W/m^2^. Cell cultures in Petri dishes immediately after UVB exposure (Image a), in cell culture flasks after being returned to the solar day/night cycle for 24 hours (Image b) and after a 20-day exposure to PAR (Image c) (control cells to the left, MC producing PCC7806 in the middle and MC non-producing PCC7005 to the right on all images).

Exposure to the higher UVB intensity (0.99 W/m^2^) induced cell granulation and massive cell lysis of both cyanobacterial strains (PCC7005 and PCC7806). Reliable cell counting was prevented by the loss of normal cell morphology, aggregation and large amounts of cellular debris (Figure 3, Panel A, Images d and g). Twentyfour hours after UVB irradiation individual cells of the MC producing PCC7806 strain were still able to preserve some original morphology (arrow in Figure 3, Panel A, Image d) while the non-producing PCC7005 cells were all in the process of disintegration (Figure 3, Panel A, Image g). None of the irradiated strains were able to resume proliferation after 20 days of cultivation under PAR. Normal cell morphology is presented in Figure 3 (Panel A, Image a) showing PCC7005 strain as an example.

### TUNEL Assay

**Figure 4:**
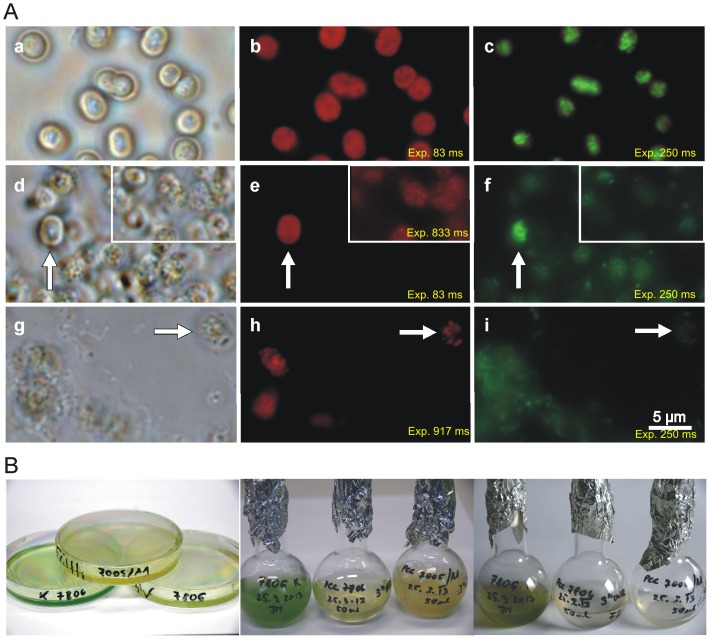
Detection of injuries induced in cyanobacteria after exposure to UVB radiation for six hours at an intensity of 0.51/m^2^. Panel A. Percentage of TUNEL-positive *M. aeruginosa* cells after UVB radiation compared with control groups. (*p<0.05, **p<0.01, ***p<0.001). Panel B. Photobleaching as a result of exposure to UVB radiation. Only MC non-producing PCC7005 Dark-acclimated cells show an increase in the percentage of cells showing no autofluorescence after UVB exposure, while a subpopulation (20%) of PAR-acclimated cells (PCC7806) almost immediately loses the signal. PAR-acclimated cells with no autofluorescence are more numerous in both MC producing strains, with an increased proportion 24 hours after exposure. Immediately after irradiation, only MC non-producing PCC7005 strain cells exhibit photobleaching (*p<0.05, **p<0.01, ***p<0.001). Panel C. Increase in percentage of cytoskeleton positive *M. aeruginosa* cells after UVB exposure. The response of PAR-acclimated MC-non-producing strain PCC7005 to UVB irradiation is immediate, while both MC-producing strains FACHB905 and PCC7806 reach the maximum of cytoskeleton labeling in 24 hours subsequent to irradiation. Only a small percentage of Dark-acclimated cells show positive immunolabeling (*p<0.05, **p<0.01, ***p<0.001). Panel D. Sedimentation of *M. aeruginosa* cells is enhanced by UVB radiation exposure. Dark-acclimated cells show faster sedimentation than PAR- acclimated. (*p<0.05, **p<0.01, ***p<0.001).

DNA fragmentation was observed immediately after exposure to UVB radiation (intensity 0.51 W/m^2^, six hours exposure) in both Dark-acclimated strains and in those exposed to PAR. DNA damage was followed in all three strains for the entire 48 hours of observation (Figure 4, Panel A).

In both MC-producing strains (FACHB905 and PCC7806), Dark-acclimated cells suffered more damage than cells maintained in the PAR environment. In contrast, in the MC non-producing PCC 7005 strain, the percentage of damaged Dark-acclimated cells remained low and stable while PAR-acclimated cells suffered major damage, with a trend towards reduced incidence with time. The highest percentage of damaged cells was observed in the Dark-acclimated PCC7806 MC-producing strain, with an increase in time reaching almost 20% at 48 hours of incubation (Figure 4, Panel A).

Due to the severe damages inflicted to cells exposed to the higher (0.99 W/m^2^) UVB intensity for three hours the assessment using TUNEL assay was not possible.

### Autofluorescence and SYBR Green Labeling

**Figure 5:**
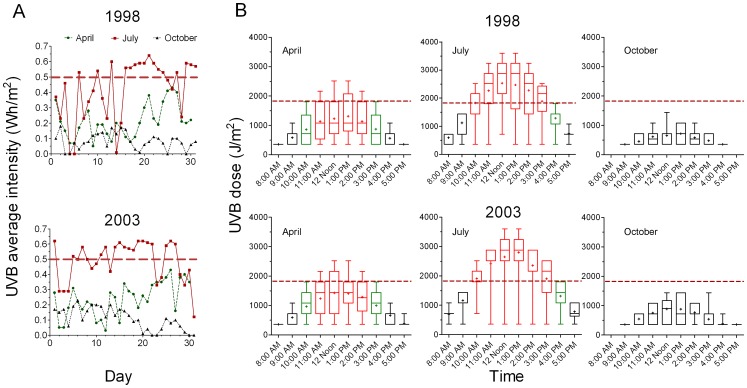
Study of *M. aeruginosa* PCC7005 PAR-acclimated cells exposed to UVB radiation for six hours at an intensity of 0.51 W/m^2^. Panel A Cells in control under light microscopy -their morphology with clear demarcation (Image a), -their homogeneous autofluorescence (Image b), and compact nucleoids (Image c). After exposure to UVB radiation, some cells lose their contrast, as observed in light microscopy, consequently becoming poorly visible with accentuated granulation (Image d, arrows a’ and b’), with reduced autofluorescence quenched to the null level (Image e, arrows a’ and b’). SYBR Green DNA staining demonstrates that, following exposure to UVB, all cells suffer some damage, depicted as blurred nucleoids. The cells that suffered more damage additionally show degradation of the genetic material (Image f, arrows a’ and b’) expressed also as a weak signal (Image f, arrow a’). Scale bar = 5 µm. Panel B. Immunolabeling of cyanobacterial cytoskeletal elements with anti-bovine α-tubulin mouse monoclonal antibody following exposure to UVB radiation. Upper row, control PCC7005 PAR-acclimated cells, PCC7005 PAR-acclimated cells exposed to UVB radiation; middle row and PCC 7806 PAR -acclimated cells exposed to UVB radiation (24 h) – lower row. Microphotographs of the same area of *M. aeruginosa* cells taken under light microscopy (Panel B, Images a, d and g), autofluorescence (Images b, e and h) and immunofluorescence (Images c, f and i). Successful immunolabeling is achieved only on heavily damaged cells with very weak or no autofluorescence. These are the cells that are barely visible under light microscopy (Image d; arrow a’), frequently granulated (arrows b’) and not detectable using autofluorescence (Image e, arrows a’ and b’; Image h, arrow c’). It can be seen that PCC7806 in the middle row (Image f; arrows a’ and b’) is immunolabeled in a way that is different from MC non-producing PCC7005 shown in Image i, lower row; arrow c’. Scale bar = 5 µm.

The three cyanobacterial strains show similar responses to UVB radiation (0.51 W/m^2^ for six hours). Cells in the control group are clearly demarcated and relatively homogeneous in their autofluorescence, with a compact nucleoid (Figure 5, Panel A, Images a, b, c). Phase contrast microscopy revealed some changes in cells exposed to UVB radiation. Accentuated granulation, with alterations at the cell wall/membrane level, was detected (Figure 5, Panel A, Image d). Their autofluorescence could be quenched to a null level (Figure 5, Panel A, Image e, arrow b’), with a blurred nucleoid indicating loosening of the genetic material (Figure 5, Panel A, Image f). Degradation of the genetic material is evident in the heavily damaged cells that are less visible under light microscopy. The SYBR Green signal is very weak and the labeled area small or even absent (Figure 5, Panel A, Image f, arrows a’ and b’).

Regardless of whether the cells in the controls are Dark- or PAR-acclimated, only a small subpopulation with a maximum of 10% is devoid of autofluorescence (Figure 4, Panel B). The percentage of UV exposed cells with no autofluorescence is high in MC non-producing PCC7005 immediately following exposure while, in both MC-producing strains, they are evident only the next day, when they exceed 50% in the PCC7806 strain. The photobleaching is not detectable in Dark-acclimated MC non-producing cells.

Only PCC7806 strain exposed to the higher UVB intensity (0.99 W/m^2^) that retained some autofluorescence was perceived at longer exposure time with a very weak and dispersed SYBR Green signal (Figure 3, Panel A, Images e and f). It is possible to detect only individual cells of the MC non-producing PCC7005 strain that retain some feeble autofluorescence but with completely degraded genetic material (Figure 3, Panel A, Images h and i) compared to control cells with homogeneous autofluorescence and compact nucleoids (Figure 3, Panel A, Images b and c).

### Cyanobacterial Cytoskeletal Framework Immunolabeling

In cyanobacterial cells that exhibit quenched autofluorescence the cytoskeletal framework becomes visible. Three pictures of the same area of control and of UVB radiation exposed cells (Figure 5, Panel B) were taken by phase contrast and by epifluorescence using G and B-2A filter sets. In the control, the cells are healthy and undamaged (Image a), their autofluorescence is homogeneous (Image b) and there is no anti-tubulin immunolabeling (Image c). On exposure to UVB radiation, some cells show damaged with immunostained cytoskeletal elements colored green-yellow, as is seen in Images f, I (Panel B). Different levels of damage can be discriminated: gradual loss of cell integrity which is reflected in their fusion with the background (Image g; arrow c’) exhibiting weak delimitation from the environment occasionally with accentuated granulation (Image d; arrow b’). The gradual loss of autofluorescence (Images e and h; arrows a’, b’ and c’) is accompanied by the emergence of immunolabeling of cytoskeletal elements (Images f and i; arrows a’, b’ and c’), while cells in control show normal morphology (Image a), homogeneous autofluorescence (Image b) and no immunolabeling (Image c in Figure 5, Panel B).

Seriously damaged cyanobacterial cells can be detected using immunolabeling of cytoskeletal elements with anti-bovine α-tubulin mouse monoclonal antibody. Dark-acclimated cells show significantly less damage than PAR-acclimated cells. There is also an essential difference between MC producing and MC non-producing strains. The MC non-producing strain PCC7005 shows immediate immunolabeling following irradiation, in contrast to the two producing strains in which the labeling reaches the maximal percentage after 24 hours. Dark acclimated cells show no significant immunolabeling of cytoskeletal elements (Figure 4, Panel C). We were not able to successfully immunostain the cells exposed to the higher (0.99 W/m^2^) UVB intensity due to excessive damage that resulted in large amounts of immunolabeled cellular debris (not shown).

### Sedimentation

The three strains in controls show similar sedimentation rates (5–15% of PAR-acclimated cells). Dark acclimatization significantly increases sedimentation of all three strains - the MC-non-producing strain (PCC7005) reaches the highest value of over 70%. Exposure to UVB radiation additionally decreases the floating capability of all strains examined. Regardless of the light conditions the most affected was the MC- non-producing PCC7005 strain in which almost the entire population (over 80%) lost their floating capability. MC-producing strains (FACHB905 and PCC7806) were also affected, although to lesser degree (Figure 4; Panel D). The sedimentation of cells exposed to higher (0.99 W/m^2^) UVB intensity could not be evaluated because of the severe changes in cell morphology (aggregation and decay).

### Environmental UVB Radiation

The calculated average all-day (from 8 AM to 5 PM) intensities presented in Figure 1 (Panel B) are lower than the average six hour intensities (the highest consecutive six hours - as the exposure in our *in vitro* experiments) to which the cyanobacterial blooms are exposed in nature. Average six hours measurements equal to or higher than 0.5 W/m^2^ (the total dose of 1800 J/m^2^ or higher on average per hour) were detected as early as mid April (spring) that persisted for even multiple (up to three) consecutive days at the end of the month (Figure 1, Panels A and B). In mid-autumn (October) the average UVB intensity no longer reached the average six hours intensity of 0.5 W/m^2^. In summer (June/July) the values frequently doubled, reaching peak values of 1.1 W/m^2^ that could be measured as early as in May (data not presented).

The hourly dose can on average, already at 10 AM in April of both chosen years (1998 with the lowest cumulative UVB dose and 2003 with the lowest cumulative UVB dose), reach (green box plots) and exceed (red box plots in Figure 1, Panel B) the biologically relevant UVB intensity of 0.5 W/m^2^ and the dose of 1800 J/m^2^ as used in *in vitro* experiments and can persist for six consecutive hours (Figure 1 Panel B). In summer the average duration of irradiance is prolonged by one to two hours of continuous UVB insolation with a two-fold higher hourly dose at midday. In mid-autumn (October) the total daily UVB irradiation falls to biologically negligible values at this altitude (Figure 1).

## Discussion

The impact of UVB radiation on cyanobacterial bloom formation and, in this respect, the role of MC production has been assessed. The damage inflicted on subcellular level of cyanobacterial cells was evaluated by visualizing the nucleoid area and cytoskeletal elements. Intensities and UVB doses (0.51 W/m^2^ for six hours = a cumulative hourly dose of 1836 J/m^2^) to which the three unicellular *Microcystis aeruginosa* strains were exposed are common in temperate latitudes as early as springtime (Figure 1), when cyanobacteria start to proliferate. In summer average hourly intensities as high as 1.2 W/m^2^ can persist for a few hours at midday (Figure 1, Panel B). With the increase in water temperature, dormant cyanobacteria are recruited from the sediment and the planktonic populations of bloom forming cyanobacterial species increase. The axenic *Microcystis* cyanobacterial species and strains cultivated *in vitro* are not directly comparable to the environmental ones since they are unicellular rather than having the natural colonial morphology, and therefore their buoyancy regulation is limited. On the other hand they display authentic damage without the interference of other organisms and allow robust statistical interpretation. As unicellular they may be more susceptible to UV radiation because of poorly expressed surface envelopes. Such unicellular morphology of cyanobacteria is frequently abundant in natural environment at the beginning of their growth season in spring.

### Evaluation of UVB Cytotoxicity

The experiments have been performed with cyanobacterial axenic strains to overcome biotic interference and to be able to assess damage indisputably. The main difference between the three strains is in their capacity for MC synthesis. PC 7005 is not capable of MC-production while PCC7806 and FACHB905 are MC-producers. Using different techniques we were able to visualize injuries over a period of 48 hours after UVB exposure. A decrease in cell number of cyanobacteria can be observed after irradiation (Figure 2) as already reported on low bacterized uni-algal cultures [15]. Cell counting itself is not enough to determine whether the decrease in cell number is due to inhibition of proliferation or to cell lysis, and if the damages are restricted to the period of exposure or prolonged into the post-irradiation phase. We do not exclude the possible inhibition of proliferation on a subpopulation of cells. The relatively high cell concentration used in our experiments (5×10^6^ cells/ml) allows mutual shading of the cells exposed to UV radiation and could consequently mitigate damages. By adopting the labeling of genetic material we have determined that the UVB exposure at an intensity of 0.5 W/m^2^ provokes serious damage from which a subpopulation of cells cannot recover but undergo apoptotic-like programmed cell death (AL-PCD) [16] (Figure 4; Panel A and Figure 5; Panel A, Image f). This can be misinterpreted as inhibition of proliferation if we overlook the cell lysis that occurs. However the subpopulation of less damaged cells is still able to proliferate and together with those capable of repair can eventually re-enter the cell cycle. As shown in Figure 2, the proliferation of the still vital subpopulation following UVB exposure must actually be faster than in the control (as in the case of PCC7806 at 48 h after exposure) in order to establish almost the same concentration as in the control (Figure 2; upper row). In contrast the MC non-producing strain PCC7005 is not able to recover over a period of 48 hours of cultivation (Figure 2; lower row). To further explain and clarify the conclusion presented above we must follow the order of precedence derived from the following experiments. Immediately after UVB irradiation almost all cyanobacterial cells show morphological changes in the nucleoid area. The staining of the DNA double helix with SYBR Green reveals a blurred nucleoid, indicating the loosening and/or destabilization of the tertiary structure in UVB exposed cells (Figure 5, Panel A; Image f) compared to control (Figure 5, Panel A; Image c). Some of the cells lose their integrity (Figure 5; Panel A, Image d), autofluorescence (Figure 5; Panel A, Image e) and normal DNA nucleoid organization, emitting weak or no SYBR Green fluorescence (Figure 5; Panel A, Image f, arrows a’ and b’). A more specific type of DNA damage is detected using the TUNEL assay (Figure 4; Panel A). This method has already been adopted successfully to pursue AL-PCD in *Microcystis* species of cyanobacteria [16]. PCD is a process that is triggered in cells unable to repair inflicted damage. The removal of such cells in bloom forming cyanobacteria results in increased fitness of the remaining population and an additional nutrient source in such heavily populated environments as cyanobacterial blooms are. Therefore still vital MC producing cyanobacteria scavenge on deteriorating ones.

The modes of AL-PCD observed in MC producing and MC non-producing strains are basically different. While a significant proportion (14%) of MC non-producing strain PCC7005 shows damage immediately after UVB irradiation, with a tendency to diminish in the following two day period, MC producing strains (FACHB905 and PCC7806) show a gradual increase in the number of TUNEL positive cells with time (Figure 4; Panel A). All such labeled cells eventually die in the process of self-destruction. The overall low immunolabeling and photobleaching of FACHB905 strain (Figure 4, Panels B and C) can be attributed to the cell fragility that results in increased amounts of immunolabeled debris (not shown).

After exposure to UVB a subpopulation of cells shows a gradual loss of autofluorescence (Figure 4; Panel B and Figure 5; Panel B, Images e and h) with consecutive immunolabelling of cytoskeletal elements (Figure 4; Panel C and Figure 5; Panel B, Images f and i), providing evidence that cytoskeletal elements become visible only in cells with reduced or null autofluorescence. These are the same cells that show major damage of the nucleoid (Figure 5; Panel A, Images e and f). Photobleaching can result from the decoupling of phycobilisomes and photosystem II reaction centers with phycobilin breakdown [17].

It has already been demonstrated that cytoskeletal elements become visible in severly damaged cells and as immunolabeled cell debris, or in stressed cells with low autofluorescence [13]. This kind of visualization enables us to detect otherwise invisible cells or cyanobacterial cell “ghosts” [13]. These are frequently smaller, as the result of cytoskeleton collapse to the degraded interior described also as honeycomb skeletal structure in the SEM microscopy study by Gumbo and Cloete (2011) [18]. Some difference in cytoskeleton labeling between the two MC producing and the MC the non-producing strain are apparent. They can be attributed to differences in the distribution of thylakoids and/or to the effectiveness of cell permeabilization (Figure 5; Panel B, Images f and i). The harmful effects of UVB radiation on the cytoskeleton have already been corroborated by experiments on inhibition of cyanobacterial motility following UV exposure [19] as well as on the degradation of cytoskeletal proteins in phytoplanktons other than cyanobacteria [20].

Although resulting in similar cumulative doses (cca. 1.1×10^4^ J/m^2^), higher UVB intensity (0.99 W/m^2^) in a shorter period of time produces significantly more lethal damages than the lower intensity (0.51 W/m^2^) protracted for an adequately longer time span (compare Figure 5 with Figure 3, Panel A).

The main reason for the quick dissolution of cells exposed to high UVB intesity (0.99 W/m^2^) is more likely due to acute physiological stress and chronic depression of key physiological processes that resulted in rapid necrosis rather than Al-PCD.

### UVB Radiation and MC Production

A reasonable explanation of the apparent link between MC-production and higher resistance to UVB radiation could lie in factors that have influenced the past development of cyanobacteria. Their evolution started in an environment hostile to life where high UV radiation was one of the major threats to complex organic molecules [21]. In parallel to this, genetic studies have provided evidence that cyanobacteria were able to produce microcystins in their early history [22], [23]. At a time when we are confronted with elevated UV irradiance due to ozone depletion, MC may regain their original role as protector against UV stress. Bloom forming cyanobacteria must cope with the negative effects of UV light by their obligatory light requirements for photosynthesis. Moderate UVB radiation results in evident oxidative stress [9]. There is scientific evidence that under such stress MC producers have a comparative advantage as MC acts as a protein-modulating metabolite and protectant, increasing the fitness of their host [11]. This protective role is possible thanks to the resistance of cyanobacterial phosphoprotein phosphatase (PPP) family to MC [24].The exposure to high UVB radiation (0.99 W/m^2^) induces irreversible damages to cyanobacteria with more pronounced effects on MC non-producing strains (Figure 3, Panels A and B).

It is experimentally confirmed that light regulates MC synthesis on two basic levels; genetic and metabolic. Transcript levels of both MC synthetase genes; *mcy B* (peptide synthase) and *mcy D* (polyketide synthase) are increased under high light and decreased when moved into the dark [25]. Both MC production and its protective binding to proteins are stimulated by light [11], [26]. However, timely delivery of unbound product to the compromised site is crucial for MC mediated protection. It can be deduced that not only the mere presence of MC, but also its production is important for MC-mediated protection. This is also supported by our experimental results showing that the Dark-acclimated MC-producing cyanobacteria, where their production is presumably stopped or slowed down, are more vulnerable to UVB exposure compared to PAR-acclimated MC-producers (Figure 4). The exact timing is still unknown but an efficacious protection is usually established before the injury takes place. It is not a coincidence that mechanisms of protection are trigerred by light since in natural environment high light intensities are usually accompanied by high intensities of biologically active UVB radiation.

### Ecological Adaptations and UV Radiation

Cyanobacteria have two basic strategies for avoiding potential UV damage. The first is the production of UV protecting pigments [12], [27] and the second, avoidance using the regulation of vertical migration. Gas vesicles enable cyanobacteria to adjust their vertical position in the water column [28]. During the vegetative period in spring, cyanobacteria are recruited from the bottom as unicellular entities or very small colonies. When cells appear on the surface their small dimensions hardly allow any vertical migration and may be exposed to irradiation for a long period of time during the day. In summer, as they grow older, the colonies become larger, capable of faster vertical migration and multiple reappearances on the water surface during the day. Not only protective pigments, the colony dimension itself can also confer UV screening due to self-shading of several layers of cells [29]. The formation of massive cell envelopes or slime layers embedding the colonies are frequently exopolysaccharide fibril structures associated with proteins [30]. Such a matrix surrounding mature colonies can also serve as an effective UV filter. Thus, microcystin production also gives in this respect an additional advantage to producing strains by promoting cell aggregation [31], [32].

It is likely that various levels of PAR and UV affect the buoyancy of cyanobacteria in different ways, depending on their action on photosynthesis. In contrast to the results of UVB on *Atrhrospira* [33], the irradiation especially of MC-non-producing *Microcystis* strains strongly diminishes their floatation capability (Figure 4, Panel D), causing damaged cyanobacteria to be lost to the bottom of deeper lakes. It must be borne in mind that all these negative effects of UVB radiation are to a certain extent mitigated by the presence of the entire light spectrum that, by photoreactivation, can reduce and repair some of UVB inflicted damages [8].

There is additional evidence that corroborates the possible advantages of MC synthesis. In spite of the metabolic burden imposed by MC synthesis, producing cyanobacteria (PCC7806) show a competitive advantage under high light conditions over MC non-producing strains [34]. In a natural *Microcystis* population, MC producing colonies are always bigger and therefore probably older than non-producing ones, proving their endurance when exposed to natural irradiance [35]. The increase in the MC-producing cyanobacteria in the bloom as the consequence of UVB radiation can lower biodiversity [36] and consequently influence food web structure and energy transfer in the waterbody.

We can conclude stratospheric ozone depletion, climate change and increased water eutrophication can act synergistically to augment the frequency and hepatotoxicity of cyanobacterial blooms.

## Conclusions

Exposure to environmental doses of UVB radiation in *in vitro* conditions provokes:

- Photobleaching effects,
- Damage to the nucleoid area and possible induction of apoptotic-like programmed cell death (AL-PCD),
- Higher proliferation rates of the undamaged subpopulation probably because of the direct use of autologous molecular material leaking from damaged cells,
- Cyanobacteria are particularly sensitive to UVB radiation in the light period of metabolic activity,
- Environmental UVB intensities may significantly influence the development and composition of cyanobacterial blooms.
- UVB irradiance may indirectly lower biodiversity by favoring MC-producing cyanobacteria.
