# Correction: UVB Radiation as a Potential Selective Factor Favoring Microcystin Producing Bloom Forming Cyanobacteria

**DOI:** 10.1371/annotation/bc9e89f0-b44c-49a7-8ea0-845a3dbd9594

**Published:** 2014-01-22

**Authors:** Yi Ding, Lirong Song, Bojan Sedmak

Multiple figures were incorrectly switched. The image currently appearing as Figure 5 belongs with the title and legend of Figure 1, the image currently appearing as Figure 1 belongs with the title and legend of Figure 2, the image currently appearing as Figure 4 belongs with the title and legend of Figure 3, the image currently appearing as Figure 2 belongs with the title and legend of Figure 4, and the image currently appearing as Figure 3 belongs with the title and legend of Figure 5. The titles and legends are in correct order.

